# Droplet microfluidics with image texture quantification for detection of rare antibiotic-resistant subpopulations from bloodstream infections

**DOI:** 10.1038/s41746-026-02808-x

**Published:** 2026-05-30

**Authors:** Sagar N. Agnihotri, Nikos Fatsis-Kavalopoulos, Emma Vikdahl, Jonas Windhager, Agustin A. Corbat, Dan I. Andersson, Maria Tenje

**Affiliations:** 1 Department of Materials Science and Engineering, Uppsala University, Uppsala, Sweden; 2 Science for Life Laboratory, Uppsala University, Uppsala, Sweden; 3 Department of Medical Biochemistry and Microbiology, Uppsala University, Uppsala, Sweden; 4 Department of Information Technology and SciLifeLab BioImage Informatics Unit, Uppsala University, Uppsala, Sweden; 5 Instituto de Física de Buenos Aires, Departamento de Física, Facultad de Ciencias Exactas y Naturales, Universidad de Buenos Aires, Buenos Aires, Argentina

**Keywords:** Biological techniques, Diseases, Microbiology

## Abstract

Heteroresistance (HR) is an antibiotic resistance phenotype characterized by the presence of rare resistant subpopulations (frequency ≈ 10^−7^ to 10^−4^) within a main susceptible bacterial population. During antibiotic exposure, these subpopulations can be enriched and cause treatment failure. Standard antibiotic susceptibility testing (AST) often fails to detect HR, and the current gold-standard population analysis profile (PAP) test is labor-intensive and time-consuming. We present a digital phenotyping approach combining droplet microfluidics with image texture to detect HR from clinical isolates, including Gram-negative (*Klebsiella pneumoniae, Pseudomonas aeruginosa, Acinetobacter baumannii*) and Gram-positive (*Staphylococcus aureus*) bacteria isolated from bloodstream infections. Our method achieves detection at subpopulation frequencies as low as 10^−6^ in 12 to 30 h, depending on bacterial species, which is faster than the PAP test, together with single-cell resolution and high-throughput. This computationally assisted microfluidic platform enables rapid and accurate identification of HR, representing a step toward targeted antibiotic therapy in critical infections.

## Introduction

Antimicrobial resistance (AMR) continues to pose one of the greatest threats to global health, contributing substantially to morbidity and mortality worldwide^1^. Traditional antimicrobial susceptibility testing (AST) remains the cornerstone for guiding therapy and reducing the risk of treatment failure^2–5^. Yet, despite the increasing sophistication and speed of new AST technologies, heteroresistance (HR), an elusive form of resistance, often escapes detection by standard AST methods^6–13^.

HR is characterized by the presence of a subpopulation of bacterial cells within an otherwise susceptible clonal population that can survive antibiotic concentrations at least eight times higher than the minimum inhibitory concentration (MIC) of the dominant population^7^. These rare resistant cells can grow during antibiotic treatment, potentially leading to therapeutic failure, and occur at frequencies typically in the range of 10^−7^ to 10^−4^ ^6,7^. The underlying mechanism of HR often involves an increased copy number of resistance genes, which, when present in single copy, are not sufficiently active to confer resistance, but when present in multiple copies they can. The high copy number state is intrinsically unstable and during routine testing of resistance, when the antibiotic selection pressure is removed, the extra gene copies are lost and not detected^6,14,15^.

Clinically significant pathogens such as *S. aureus*, *K. pneumoniae*, *E. coli*, *P. aeruginosa*, and *A. baumannii*, responsible for many types of infections, including bloodstream, pneumonia, and urinary tract infections, have all been shown to exhibit HR phenotypes^16^. These species also dominate the WHO priority pathogen list due to their increasing resistance to last-resort antibiotics^17^. Although prevalence estimates of HR vary across studies, a growing body of evidence confirms its widespread occurrence and clinical relevance across these critical pathogens and antibiotic classes^12,13,18–30^. Despite its importance, HR remains underdiagnosed because the current gold standard, the population analysis profile (PAP) test, is labor-intensive and time-consuming and therefore not used. Moreover, routine AST often fails to detect HR subpopulations due to a lack of sensitivity^10,31,32^. This diagnostic blind spot underscores an urgent need for innovative technologies capable of detecting HR rapidly and reliably.

Droplet-based microfluidics offers a promising solution to HR diagnostics. By encapsulating bacterial populations within nanoliter or femtoliter droplets, this technology enables high-throughput testing at the single-cell or microcolony level^33–35^. In this approach, aqueous or hydrogel-based^36^ droplets are generated within an oil phase, where they can be manipulated on-chip using active^37–39^ or passive methods^40^ for diverse biological applications^41–43^. Although a few studies have explored droplet microfluidics for HR detection^44,45^, most rely on Poisson-distributed single-cell encapsulation, leaving the majority of droplets empty. For subpopulations that occur as rarely as 10^−6^–10^−7^, such strategies demand millions of droplets to achieve statistical detection, making them impractical for clinical use.

In previous work from our labs^46^, we addressed these limitations by developing a droplet shrinkage–based method to detect HR in *E. coli* bloodstream isolates. Although highly suitable for HR detection in *E. coli*, this approach depended on the specific growth medium composition and bacterial metabolism, making it less effective for Gram-positive species such as *S. aureus*, especially when tested in Mueller–Hinton (MH) broth, which limits the method’s reliability for detecting HR in Gram-positive clinical isolates. To overcome these constraints, we introduce a new detection method that integrates droplet microfluidics with image texture analysis, a technique capable of detecting HR in both Gram-positive and Gram-negative bacteria, faster than our earlier shrinkage-based approach and the gold-standard PAP test. Our new proposed image-based texture analysis provides a medium- and species-independent readout that can be applied to all species under standard conditions. In addition, even in cases where shrinkage occurs, we observe that the shrinkage typically appears 1–2 h later than the texture change, meaning our new method provides an earlier indication of growth.

Image texture analysis quantifies the spatial arrangement of pixel intensities in microscopic images, revealing patterns that correspond to underlying biological structures. For instance, a smooth, uniform texture might represent a homogeneous background, while a coarse or granular texture could signify the presence of cells, colonies, or other biological structures. The primary goal of texture analysis is to quantify these spatial patterns using various statistical methods and algorithms (e.g., Gray-Level Co-occurrence Matrix) into numerical data, enabling automated and objective classification of complex biological images^47^. Texture analysis has previously been applied to detect microbial contamination in water, classify cell types, and monitor growth kinetics of encapsulated microorganisms^48^.

In this study, we combine texture-based image analysis with droplet microfluidics to detect rare resistant bacterial subpopulations in clinical isolates obtained from bloodstream infections. By distinguishing droplets containing growing bacterial subpopulations from empty ones, this integrated approach provides a rapid, sensitive, and scalable method to uncover HR in both Gram-negative and Gram-positive pathogens, paving the way for its implementation in clinical diagnostics.

## Results

**Fig. 1:**
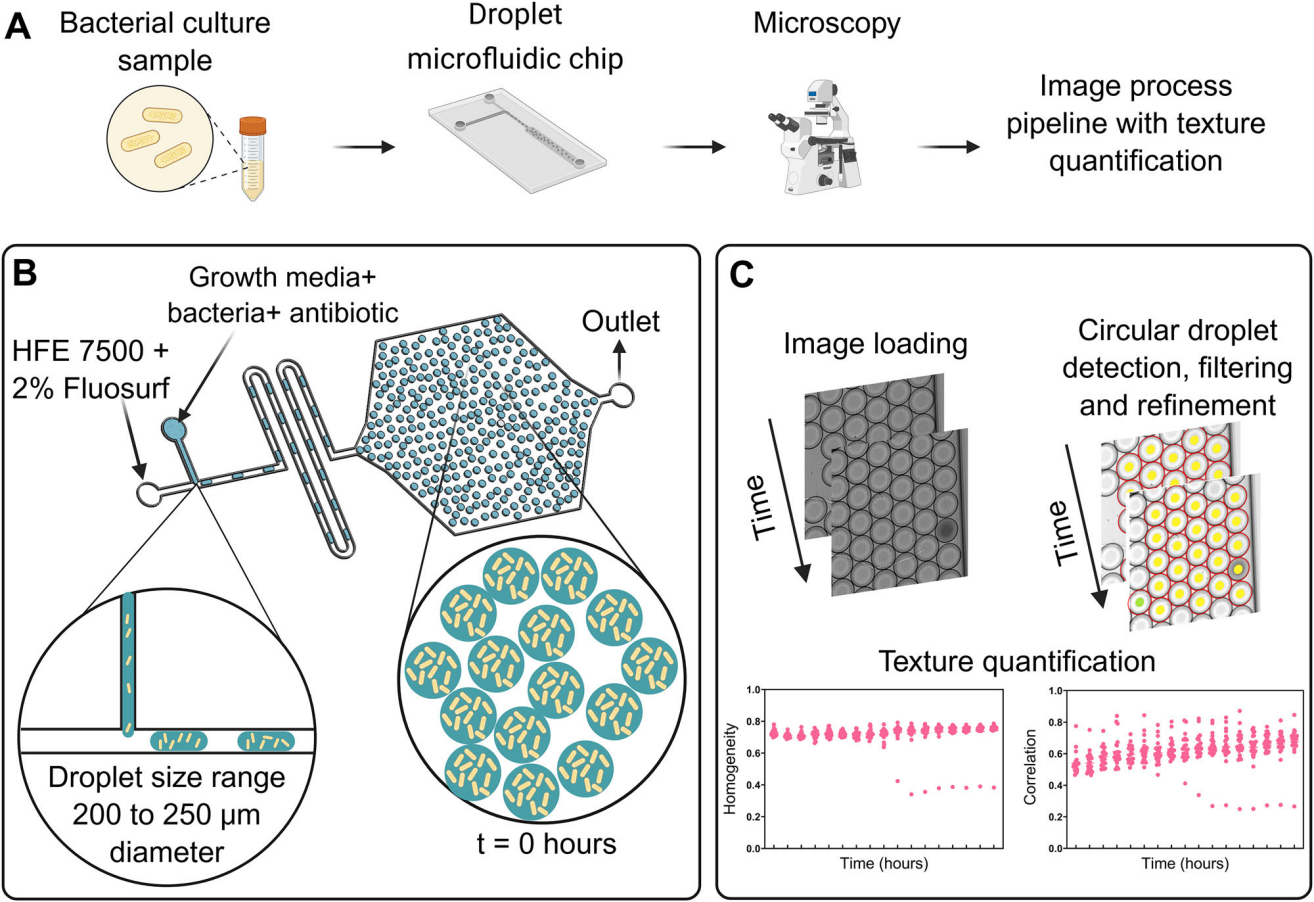
Schematic representation of the HR detection in Gram-negative and Gram-positive bacteria from bloodstream infection. **A** The main steps involved in the workflow from overnight bacterial culture to the image process pipeline, involving texture quantification. **B** Illustration of the microfluidic geometry for droplet generation and storage involving HFE-7500 with 2% Fluosurf as a continuous phase and growth medium with bacteria and the antibiotic of choice as the dispersed phase. **C** Main steps involving the texture quantification from the time-lapse image sequence. Each dot in a plot of homogeneity and correlation represents the value of texture quantification of every droplet analyzed. Created in BioRender. Agnihotri, S. (2026) https://BioRender.com/mulwjsl.

The overall workflow for HR detection is shown in Fig. 1A. The process begins with overnight liquid cultures of the bacterial species of interest in a growth medium. For Gram-negative bacteria (*K. pneumoniae*, *P. aeruginosa*, and *A. baumannii*), Mueller Hinton Broth (MHB) support the growth of rare resistant subpopulations, was used. In contrast, for *S. aureus*, the emergence of resistant subpopulations was slow in MHB compared with Brain Heart Infusion (BHI) medium, and therefore BHI was instead used for all *S. aureus* experiments.

Following overnight culture, the bacterial suspension was diluted to the desired concentration and used as the dispersed phase, while HFE oil containing 2% Fluosurf was used as the continuous phase for droplet generation, as shown in Fig. 1B. Additional details of the microfluidic setup and operating conditions are provided in the *Methods* section. The generated droplets were retained within the same microfluidic chip and incubated at 37 °C in a stage-top incubator, with images acquired at regular time intervals to monitor bacterial growth.

The acquired images were analyzed using a custom image-processing pipeline (Fig. 1C) designed to quantify droplet-level mean intensities and gray-level co-occurrence matrix (GLCM) texture properties, including dissimilarity, homogeneity, angular second moment (ASM), energy, and correlation. Among these, *homogeneity* and *correlation* most effectively captured the emergence and growth dynamics of rare antibiotic-resistant subpopulations, and they were therefore chosen after manual qualitative inspection and used as quantitative markers of bacterial growth. Results for each bacterial species are presented in the subsequent sections.

Detection of *A. baumannii* HR: To detect HR in *A. baumannii* against amikacin, we used two different clinical isolates: a non-HR susceptible strain (DA33414) and an HR strain (DA33098) (as confirmed by PAP tests). Each strain was tested in three biological replicates (N1, N2, and N3). The initial bacterial concentration was maintained at 5 × 10⁸ CFU/mL, and the amikacin concentration at 24 mg/L. At this bacterial concentration, each droplet encapsulated thousands of bacteria. The average droplet diameter, corresponding droplet volume, and estimated encapsulation per droplet for each experiment are summarized in Supplementary Table 1. Images were acquired every two hours over a total duration of 24 h.

**Fig. 2:**
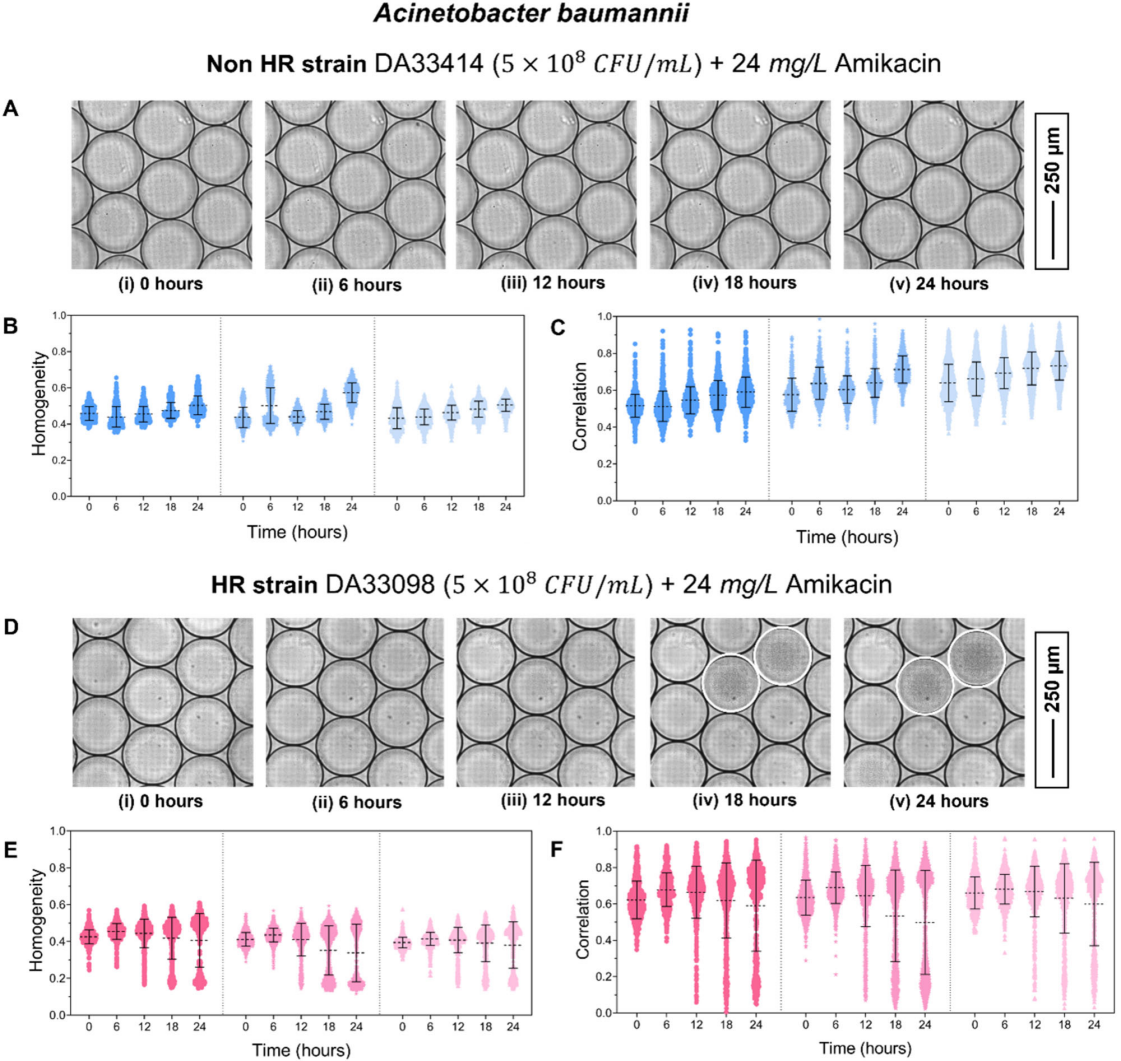
Detection of A. baumannii HR. **A**, (**i**–**v**) Image sequence showing the non-HR *A. baumannii* strain DA33414 exposed to 24 mg/L amikacin at 0, 6, 12, 18, and 24 h. No bacterial growth is observed in any droplets. **B**, **C** Texture parameters homogeneity (**B**) and correlation (**C**) plotted over time for the non-HR strain. Data from three biological replicates are shown, separated by dotted lines. **D**, **i**–**v** Image sequence showing the HR strain DA33098 exposed to 24 mg/L amikacin at 0, 6, 12, 18, and 24 h. Droplets exhibiting bacterial growth are outlined in white. Texture parameters homogeneity (**E**) and correlation (**F**) are plotted over time for the HR strain. Data from three biological replicates are shown, separated by dotted lines.

Figure 2A(i–v) shows an image sequence for the non-HR susceptible strain exposed to 24 mg/L amikacin. No bacterial growth was observed in any droplets over 24 h, indicating the absence of rare antibiotic-resistant subpopulations in this strain. To quantify droplet image features, we analyzed two texture parameters: homogeneity (Fig. 2B) and correlation (Fig. 2C). Both parameters remained constant throughout the experiment. The same analysis was performed for the HR strain. Figure 2D (i–v) shows an image sequence for this strain under identical conditions. Droplets exhibiting bacterial growth are marked with white borders. The corresponding texture analyses for homogeneity (Fig. 2E) and correlation (Fig. 2F) show a marked decrease in both parameters in droplets with bacterial growth across all replicates. At the final time point, a clear distinction is evident between droplets with and without growth based on texture values. Some droplets exhibited partial or limited bacterial growth, appearing intermediate between the two categories; a magnified image of each type of droplet (with growth, without growth, and partial growth) in general is shown in Supplementary Fig. 1.

To classify droplets as either growth or no-growth, the cutoff criteria for classifying growth vs. non-growth are determined. In this case, the lowest value of homogeneity and correlation at 0 h in each experiment is used as the cutoff criterion for classifying and calculating the resistant subpopulation frequency at the 24-h timepoint.

Finally, we calculated the resistant subpopulation frequency for the HR strain based on the total number of droplets analyzed, the number of droplets showing bacterial growth, and the estimated bacterial encapsulation per droplet (Supplementary Table 1). The detailed model used for calculating the resistant subpopulation frequency is provided in the Supplementary Note 1. The results are summarized in Table 1, where *N*_d_ and *N*_G_ represent the number of droplets analyzed and the number of droplets showing bacterial growth according to the homogeneity and correlation cutoff value. The frequencies estimated using homogeneity and correlation were in close agreement, with only minor variations attributable to the cutoff criteria employed.

**Table 1:** Data showing the number of droplets analysed (N_d_), the number of droplets showing growth according to the homogeneity and correlation cutoff value (N_G_), and calculated subpopulation frequency for A

	N_d_	N_G_ (Homogeneity)	Subpopulation Frequency	N_G_ (Correlation)	Subpopulation Frequency
Non-HR strain DA33414 (5 × 10^8^ CFU/mL) in MH broth + 24 mg/L Amikacin					
N_1_	608	0	0	0	0
N_2_	780	0	0	0	0
N_3_	1299	0	0	0	0
HR strain DA33098 (5 × 10^8^ CFU/mL) in MH broth + 24 mg/L Amikacin					
N_1_	710	203	1.1 × 10^−4^	176	9.1 × 10^−5^
N_2_	1130	522	1.9 × 10^−4^	430	1.5 × 10^−4^
N_3_	717	214	1.2 × 10^−4^	197	1.1 × 10^−4^

baumannii non-HR (DA33414) and HR (DA33098) strain against 24 mg/L amikacin.

**Fig. 3:**
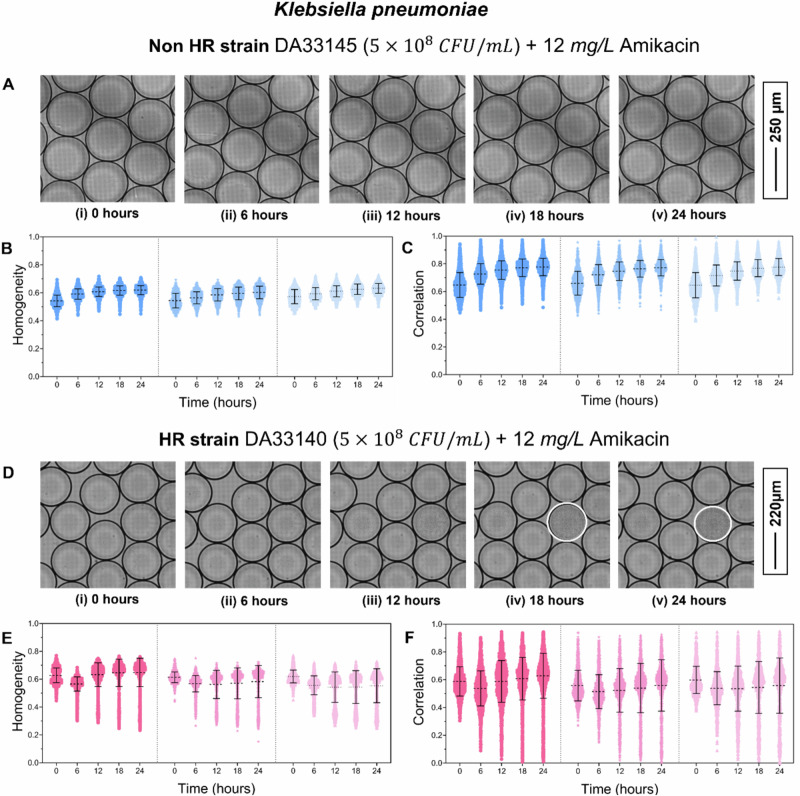
Detection of K. pneumoniae HR. **A**, (**i**–**v**) Image sequence showing the non-HR *K pneumoniae* strain DA33145 exposed to 12 mg/L amikacin at 0, 6, 12, 18, and 24 h. No bacterial growth is observed in any droplets. Texture parameters homogeneity (**B**) and correlation (**C**) plotted over time for the non-HR strain. Data from three biological replicates are shown, separated by dotted lines. **D**, **i**–**v** Image sequence showing the HR strain DA33140 exposed to 12 mg/L amikacin at 0, 6, 12, 18, and 24 h. Droplets exhibiting bacterial growth are outlined in white. Texture parameters homogeneity (**E**) and correlation (**F**) are plotted over time for the HR strain. Data from three biological replicates are shown, separated by dotted lines.

Detection of *K. pneumoniae* HR: To assess HR detection in *K. pneumoniae*, we tested a non-HR susceptible strain (DA33145) and an HR strain (DA33140) at 5 × 10⁸ CFU/mL in the presence of 12 mg/L amikacin. Each strain was analyzed in three biological replicates. Droplet size, volume, and bacterial encapsulation are provided in Table S1. No bacterial growth was observed in the non-HR strain over 24 h (Fig. 3A, i–v), and texture parameters, including homogeneity (Fig. 3B) and correlation (Fig. 3C), remained stable.

In the HR strain, droplets with bacterial growth were clearly distinguishable (Fig. 3D, i–v, outlined in white), and homogeneity and correlation decreased in these droplets across all replicates (Fig. 3E, F). Partial growth droplets exhibited intermediate texture values (Supplementary Fig. 1). Using the minimum homogeneity and correlation values from each experiment at 0 h as cutoffs, resistant subpopulation frequencies were calculated from the number of growth-positive droplets and estimated bacterial encapsulation per droplet (Table 2). Frequencies derived from both texture parameters were in close agreement.

**Table 2:** Data showing the number of droplets analysed (N_d_), the number of droplets showing growth according to the homogeneity and correlation cutoff value (N_G_), and calculated subpopulation frequency for K

	N_d_	N_G_ (Homogeneity)	Subpopulation Frequency	N_G_ (Correlation)	Subpopulation Frequency
Non-HR strain DA33145 (5 × 10^8^ CFU/mL) in MH broth + 12 mg/L Amikacin					
N_1_	1860	0	0	0	0
N_2_	1751	0	0	0	0
N_3_	1529	0	0	0	0
HR strain DA33140 (5 × 10^8^ CFU/mL) in MH broth + 12 mg/L Amikacin					
N_1_	1725	141	3.4 × 10^−5^	112	2.7 × 10^−5^
N_2_	1453	233	8.8 × 10^−5^	165	6.2 × 10^−5^
N_3_	1124	264	1.3 × 10^−4^	207	1 × 10^−4^

pneumoniae non-HR (DA33145) and HR (DA33140) strain against 12 mg/L amikacin.

Detection of *P. aeruginosa* HR: HR in *P. aeruginosa* was examined using a non-HR susceptible strain (DA69786) and an HR strain (DA69806). The initial bacterial concentration was set to 10⁸ CFU/mL, and droplets were exposed to 8 mg/L meropenem. Meropenem can be degraded and undergoes spontaneous hydrolysis in aqueous solution; hence, the initial loading was reduced to a few hundred bacteria per droplet instead of thousands. Each strain was analyzed in three biological replicates, and droplet characteristics, including size, volume, and encapsulation, are listed in Table S1.

**Fig. 4:**
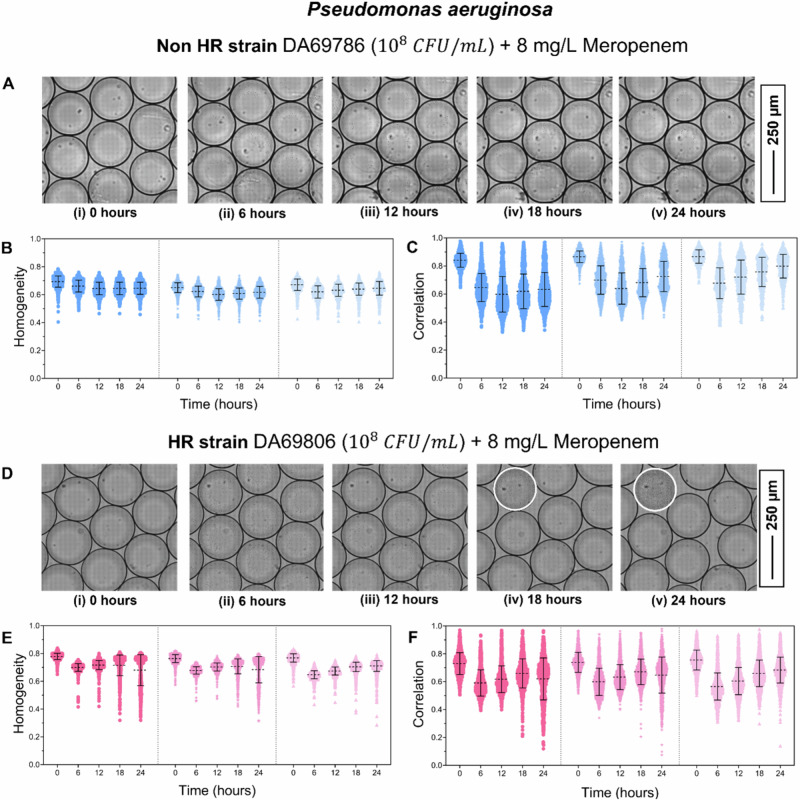
Detection of P. aeruginosa HR. **A**, **i**–**v** Image sequence showing the non-HR *P. aeruginosa* strain DA69786 exposed to 8 mg/L meropenem at 0, 6, 12, 18, and 24 h. No bacterial growth is observed in any droplets. Texture parameters homogeneity (**B**) and correlation (**C**) plotted over time for the non-HR strain. Data from three biological replicates are shown, separated by dotted lines. **D**, **i**–**v** Image sequence showing the HR strain DA69806 exposed to 8 mg/L meropenem at 0, 6, 12, 18, and 24 h. Droplets exhibiting bacterial growth are outlined in white. Texture parameters homogeneity (**E**) and correlation (**F**) are plotted over time for the HR strain. Data from three biological replicates are shown, separated by dotted lines.

For the non-HR strain, no droplets exhibited bacterial growth during the 24-hour incubation (Fig. 4A, i–v). The texture parameters homogeneity (Fig. 4B) and correlation (Fig. 4C) showed minimum values of 0.40 and 0.34, respectively. Unlike the other species examined, droplets without bacterial growth in *P. aeruginosa* did not display a clean background; instead, small dot-like residual structures appeared after antibiotic action, likely corresponding to bacterial debris or lysed cells. A magnified view of these features is shown in Fig. S2. Consequently, a slight reduction in texture parameters was observed even in the non-HR strain, reflecting image artifacts rather than true bacterial growth, specifically for correlation. In all three experiments of the non-HR strain, the correlation decreases until 6 hand then stabilizes. Hence, the lowest value of correlation at 6 h was used as the cutoff criterion in case of correlation, while for homogeneity lowest value at 0 h was used as the cutoff criterion.

The HR strain displayed distinct droplets with visible bacterial growth (Fig. 4D, i–v, white outlines). Texture analysis revealed consistent declines in both homogeneity and correlation in these droplets across all biological replicates (Fig. 4E, F). Even in the case of HR strain, the droplet without subpopulation growth showed small dot-like residual structures after antibiotic action. Based on the number of growth-positive droplets and the estimated bacterial encapsulation per droplet, the resistant subpopulation frequency was determined (Table 3).

**Table 3:** Data showing the number of droplets analysed (N_d_), the number of droplets showing growth according to the homogeneity and correlation cutoff value (N_G_), and calculated subpopulation frequency for P

	N_d_	N_G_ (Homogeneity)	Subpopulation Frequency	N_G_ (Correlation)	Subpopulation Frequency
Non-HR strain DA69786 (10^8^ CFU/mL) in MH broth + 8 mg/L Meropenem					
N_1_	1447	0	0	0	0
N_2_	1256	0	0	0	0
N_3_	612	0	0	0	0
HR strain DA69806 (10^8^ CFU/mL) in MH broth + 8 mg/L Meropenem					
N_1_	1079	368	7.8 × 10^−4^	119	2.5 × 10^−^^4^
N_2_	1397	244	3.3 × 10^−4^	84	1.1 × 10^−4^
N_3_	1459	15	1.9 × 10^−5^	4	5.2 × 10^−6^

aeruginosa non-HR (DA69786) and HR (DA69806) strain against 8 mg/L meropenem.

**Fig. 5:**
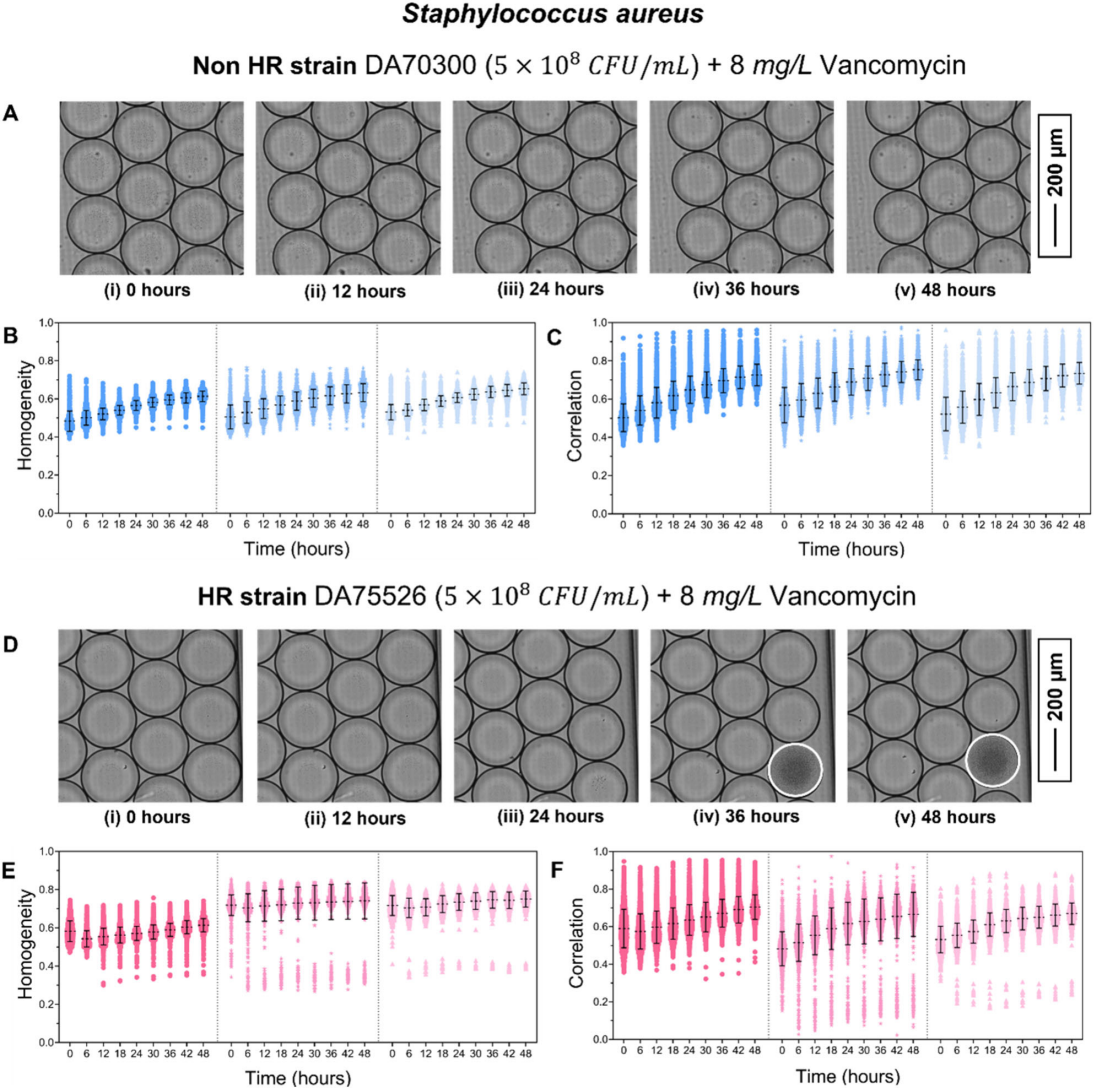
Detection of S. aureus HR. **A**, **i**–**v** Image sequence showing the non-HR *S. aureus* strain DA70300 exposed to 8 mg/L vancomycin at 0, 12, 24,36 and 48 h. No bacterial growth is observed in any droplets. Texture parameters homogeneity (**B**) and correlation (**C**)) plotted over time for the non-HR strain. Data from three biological replicates are shown, separated by dotted lines. **D**, **i**–**v** Image sequence showing the HR strain DA75526 exposed to 8 mg/L vancomycin at 0, 12, 24,36 and 48 h. Droplets exhibiting bacterial growth are outlined in white. Texture parameters homogeneity (**E**) and correlation (**F**) are plotted over time for the HR strain. Data from three biological replicates are shown, separated by dotted lines.

Detection of *S. aureus* HR: To determine HR in *S. aureus*, we tested a non-HR susceptible strain (DA70300) and an HR strain (DA75526) at an initial concentration of 5 × 10⁸ CFU/mL in the presence of 8 mg/L vancomycin. Brain Heart Infusion (BHI) medium was used instead of Mueller–Hinton (MH) broth, as rare resistant subpopulations did not grow consistently in MH even after 72 h. We also observed variability when using BHI medium older than three days; therefore, all experiments were initiated within 2–3 days of media preparation. Under these conditions, resistant subpopulations appeared in all experiments within 48 h, and the total experimental duration was set accordingly. Each strain was analyzed in three biological replicates. Droplet size, volume, and estimated bacterial encapsulation are summarized in Table S1. No bacterial growth was observed in the non-HR strain during the 48 h (Fig. 5A, i–v). Correspondingly, texture parameters homogeneity (Fig. 5B) and correlation (Fig. 5C) remained stable.

In contrast, droplets containing bacterial growth were clearly visible in the HR strain (Fig. 5D, i–v, outlined in white). Both homogeneity and correlation decreased in these droplets across all biological replicates (Fig. 5E, F). Notably, resistant subpopulations appeared at different time points, with some droplets showing growth as early as 12–15 h and others emerging after approximately 30 h, indicating temporal heterogeneity in HR.

Resistant subpopulation frequencies were calculated using the minimum homogeneity and correlation values from each experiment at 0 h as cutoff thresholds, along with the number of growth-positive droplets and the estimated bacterial encapsulation per droplet (Table 4). Frequencies derived from both texture parameters were in close agreement.

**Table 4:** Data showing the number of droplets analysed (N_d_), the number of droplets showing growth according to the homogeneity and correlation cutoff value (N_G_), and calculated subpopulation frequency for S

	N_d_	N_G_ (Homogeneity)	Subpopulation Frequency	N_G_ (Correlation)	Subpopulation Frequency
Non-HR strain DA70300 (5 × 10^8^ CFU/mL) in BHI media + 8 mg/L Vancomycin					
N_1_	1227	0	0	0	0
N_2_	1597	0	0	0	0
N_3_	1829	0	0	0	0
HR strain DA75526 (5 × 10^8^ CFU/mL) in BHI media + 8 mg/L Vancomycin					
N_1_	1915	11	2.6 × 10^−6^	3	7.04 × 10^−7^
N_2_	1568	56	1.7 × 10^−5^	74	2.2 × 10^−5^
N_3_	1287	10	3.6 × 10^−6^	10	4.3 × 10^−6^

aureus non-HR (DA70300) and HR (DA75526) strain against 8 mg/L vancomycin.

Performance of image texture quantification: To evaluate the performance of texture-based quantification, we analyzed subsampled image datasets using homogeneity and correlation features and compared the model outputs with manually verified ground-truth labels. For non-HR strains in each bacterial species, ten time-lapse image sequences (each image contains 13 or 17 timepoints, depending on the species and total imaging time) were selected randomly from the experimental dataset. For HR strains, ten time-lapse image sequences were chosen such that each contained at least one droplet exhibiting bacterial growth over time. The final time-point image was then manually inspected to determine the growth status of the droplets (ground-truth), which were compared to the automatic classification by the feature-based cutoff criterion defined for each experiment (prediction).

**Fig. 6:**
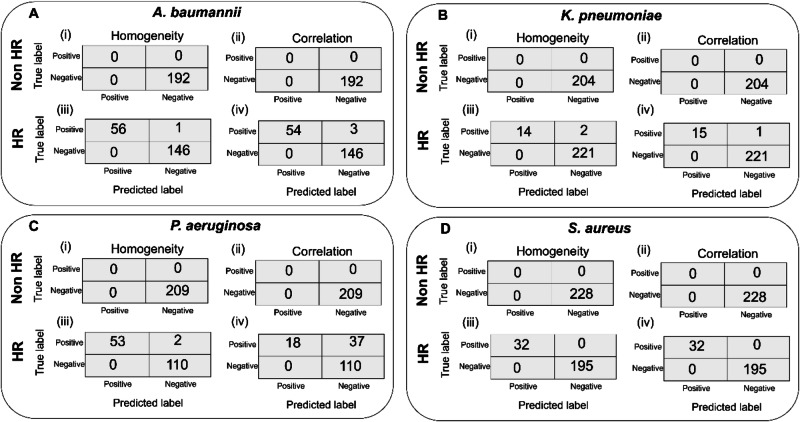
Performance of image texture quantification. Confusion matrices showing the performance of homogeneity and correlation features, evaluated using down-sampled image data, for distinguishing true and predicted labels across different bacterial species and HR. For **A** *A. baumannii*, results are shown for non-HR strains using homogeneity (i) and correlation (ii), and for HR strains using homogeneity (iii) and correlation (iv). **B** For *K. pneumoniae*, non-HR strain performance is shown using homogeneity (i) and correlation (ii), and HR strain performance using homogeneity (iii) and correlation (iv). **C** Similarly, for *P. aeruginosa*, non-HR strains are evaluated using homogeneity (i) and correlation (ii), and HR strains using homogeneity (iii) and correlation (iv). **D** Finally, for *S. aureus*, the non-HR strain results are shown using homogeneity (i) and correlation (ii), and the HR strain results using homogeneity (iii) and correlation (iv).

The confusion matrices for non-HR and HR strains of *A. baumannii* are shown in Fig. 6A (i–iv). Both homogeneity and correlation correctly detected the non-HR strain with perfect agreement with the ground-truth (no false positives or false negatives). For the HR strain, out of 203 droplets, homogeneity and correlation yielded 1 and 3 false negatives, respectively but no false positives. Similar results were observed for the other species: *K. pneumoniae* (Fig. 6B), P. *aeruginosa* (Fig. 6C), and *S. aureus* (Fig. 6D). In all species, both homogeneity and correlation detected non-HR strains flawlessly.

For HR strains, homogeneity identified *K. pneumoniae*, *P. aeruginosa*, and *S. aureus* with high precision, resulting in 2, 2, and 0 false negatives out of 236, 165, and 227 droplets, respectively, with no false positives. Correlation also detected HR strains accurately for *K. pneumoniae* and *S. aureus*, with 1 and 0 false negatives out of 237 and 227 droplets, respectively. However, correlation performed poorly for *P. aeruginosa*, yielding 37 false negatives out of 165 droplets. This reduced performance arises from the presence of small dot-like residual structures remaining after antibiotic exposure, likely corresponding to bacterial debris or lysed cells. Additionally, we have also provided accuracy, precision, recall, and F1 score to quantify the performance of texture feature analysis for each strain that we have analyzed, as shown in the Table 5. These evaluation parameters are calculated using true positive (TP), false negative (FN), false positive (FP), and true negative (TN). The accuracy is defined as (TP + TN)/Total, precision as (TP)/(TP + FP), recall as (TP)/(TP + FN), and F1 score as (2×precision×recall)/(precision + recall).

**Table 5:** The evaluation parameter accuracy, precision, recall, and F1 score, are shown for all the strains to quantify the performance of texture feature homogeneity(H) and correlation (C)

	Accuracy (%)		Precision (%)		Recall (%)		F1 score (%)	
	H	C	H	C	H	C	H	C
***A. baumannii***								
Non-HR	100	100	-	-	-	-	-	-
HR	99.5	98.5	100	100	98.2	94.7	99.1	97.3
***K. pneumoniae***								
Non-HR	100	100	-	-	-	-	-	-
HR	99.2	99.6	100	100	87.5	93.7	93.3	96.7
***P. aeruginosa***								
Non-HR	100	100	-	-	-	-	-	-
HR	98.8	77.6	100	100	96.4	32.7	98.2	49.3
***S. aureus***								
Non-HR	100	100	-	-	-	-	-	-
HR	100	100	100	100	100	100	100	100

Collectively, these results demonstrate that texture-based quantification exhibits strong predictive agreement with manually verified labels across diverse bacterial species. This shows its potential as a rapid, label-free approach for reliable identification of HR. However, care must be taken when using the correlation for *P. aeruginosa* strains that exhibit small residual structures after antibiotic exposure.

## Discussion

Our study demonstrates that integrating droplet-based microfluidics with quantitative image texture analysis provides a robust approach for detecting HR in bacteria isolated from bloodstream infections. Using this method, we successfully identified HR in *A. baumannii*, *K. pneumoniae*, *P. aeruginosa*, and *S. aureus*. The total time required for imaging varied depending on the bacterial species: 24 h for Gram-negative bacteria and 48 h for Gram-positive bacteria. Based on the current results (see FigS. 2–5), the emergence of antibiotic-resistant subpopulations was detectable as early as 12 h for *A. baumannii*, 6–12 h for *K. pneumoniae*, 12 h for *P. aeruginosa*, and 15–30 h for *S. aureus*.

Conventional AST has recently been complemented by emerging microscale approaches such as confined microwell platforms, which enable rapid growth readouts at the single-cell level^49,50^. For HR detection, several strategies rely on single-cell encapsulation in droplets to directly quantify phenotypic heterogeneity within bacterial populations^44,45^. However, when droplet assays are operated in the single-cell regime, the encapsulation follows Poisson statistics, leading to a high fraction of empty droplets. Consequently, to reliably detect rare HR subpopulations at clinically relevant frequencies (e.g., ≈10^−6^), these approaches may require millions of droplets. In contrast, a recently reported droplet shrinkage-based HR detection strategy enables the detection of rare resistant subpopulations using only a few hundred droplets (around 300), but was demonstrated primarily for Gram-negative bacteria^46^. The method presented here maintains the advantage of requiring only around 300 droplets while extending applicability to both Gram-negative and Gram-positive bacteria.

Automated image texture quantification specifically using the homogeneity and correlation parameters enabled differentiation between droplets with and without bacterial growth, allowing accurate estimation of the frequency of resistant subpopulations. The platform achieved a detection sensitivity of one resistant bacterium among one million susceptible cells. With modest scaling of the device size, droplet volume, and droplet throughput, this limit could be further improved to one in ten million. Compared with previously reported microfluidic-based approaches for HR detection, our method provides both superior sensitivity and broad applicability to clinically relevant Gram-negative and Gram-positive pathogens. These results underscore the potential of image texture–based droplet microfluidics as a universal platform for investigating phenotypic resistance heterogeneity.

To benchmark our method against a gold standard for HR detection, we compared results with the population analysis profile (PAP) test. Table 6 summarizes the average subpopulation frequencies (*n* = 3) determined by homogeneity and correlation parameters alongside PAP results. The calculated subpopulation frequency with both the texture parameters matches closely with that of the PAP test results. There are two main limitations of our assay. First, even though it is still significantly faster than the PAP test, which requires 2–3 days, readout might require 1 day. The second is that antibiotic-specific modes of action, particularly those associated with β-lactams, can give rise to non-growing residual structures (as observed in *P. aeruginosa*) that affect certain texture metrics, such as correlation. We emphasize that this represents a limitation specific to image interpretation rather than HR detection itself and a systematic characterization of such residual phenotypes is an interesting direction for future work, along with incorporating multiple antibiotic concentrations per isolate to more closely emulate full PAP profiles or breakpoint-based stratification if required for specific clinical applications. A further improvement could be achieved through droplet immobilization strategies, such as thermosetting oils that enable stable droplet arrays, which have been reported^51,52^ and could provide a practical solution for consistent droplet tracking and temporal feature extraction.

**Table 6:** Comparison between the average resistant subpopulation frequency calculated from image texture quantification and that determined by the population analysis profile (PAP) test

Strain Information	Comment	Average Subpopulation frequency with homogeneity	Average Subpopulation frequency with correlation	Subpopulation frequency with the PAP test
*A. baumannii* in MHB + 24 mg/L amikacin				
DA33414	Non-HR	0	0	0
DA33098	HR	1.4 × 10^−4^	1.2 × 10^−4^	>10^−4^
*K*. *pneumoniae* in MHB + 12 mg/L amikacin				
DA33145	Non-HR	0	0	0
DA33140	HR	8.4 × 10^−5^	6.3 × 10^−5^	10.3 × 10^−5^
*P. aeruginosa* in MHB + 8 mg/L meropenem				
DA69786	Non-HR	0	0	0
DA69806	HR	3.8 × 10^−4^	1.2 × 10^−4^	2.3 × 10^−4^
*S. aureus* in BHI + 8 mg/L vancomycin				
DA70300	Non-HR	0	0	0
DA75526	HR	2.1 × 10^−6^	9 × 10^−6^	1.2 × 10^−6^

When it comes to the cost of a PAP assay for detecting HR, the method is intrinsically resource-intensive. For a single isolate tested against one antibiotic, PAP typically requires preparation of a dilution series across multiple agar plates, often 12 to 20 plates, to adequately span the relevant concentration range and include technical replicates and controls. Based on a typical European laboratory, this corresponds to consumables per isolate per antibiotic, including agar plates, antibiotics, and routine laboratory disposables. However, the dominant cost of PAP lies in personnel time. Serial dilutions, plating, labeling, incubation handling, and manual colony counting across multiple plates typically require between 1.5 and 3 h of hands-on technician time per isolate. In contrast, the droplet microfluidic platform presented here shifts the cost structure away from labor-intensive manual handling toward automated readout. For a single isolate tested against one antibiotic, consumable costs include a single-use microfluidic chip, oil and surfactant, culture media, antibiotic, and standard disposables. Hands-on time is largely limited to culture preparation, chip loading, and basic quality control of the automated image analysis output, typically requiring less than 1 hour. Importantly, the labor advantage of the microfluidic platform increases further with batching, as image acquisition and texture-based classification scale computationally rather than linearly with manual effort. In contrast, PAP labor scales directly with the number of plates and colonies that must be enumerated, making it poorly suited for high-throughput or routine use. While routine clinical AST methods are less expensive than PAP, they lack the sensitivity to detect rare HR subpopulations and therefore do not address the same clinical question. Taken together, when consumables and personnel time are both considered, the droplet microfluidic platform is approximately 1.5 to 2.5-fold less expensive per isolate per antibiotic than the PAP assay under conservative assumptions, while also providing a substantially shorter turnaround time and automated readout. This reduction in cost and labor, combined with improved scalability, supports the potential of this approach for broader clinical adoption as a practical alternative to PAP for HR detection.

Beyond direct assay costs, time to result represents a substantial and often underestimated economic factor in clinical care. Delayed detection of HR can lead to prolonged use of ineffective antibiotic regimens, delayed escalation or optimization of therapy, and extended hospital stays, all of which carry high downstream costs for healthcare systems. In bloodstream infections, even modest delays in appropriate antimicrobial therapy are associated with increased length of stay, higher rates of complications, and increased use of intensive care resources^53,54^. By reducing the time to actionable results from several days, as required for PAP, to 6 to 30 h, the droplet microfluidic platform has the potential to enable earlier treatment optimization. This earlier intervention can translate into reduced antibiotic exposure, shorter hospitalizations, and lower overall healthcare costs, benefits that are not captured by per-test pricing alone but are critical for hospital-level cost effectiveness and clinical decision-making.

In conclusion, this work establishes a foundation for a new class of digital diagnostic tools that combine droplet microfluidics with computational image texture analysis to detect bacterial HR with a sensitivity that exceeds standard antibiotic susceptibility testing. By coupling microscale experimental precision with imaging and texture analysis, the platform bridges experimental microbiology and digital medicine. Beyond enabling sensitive detection of rare resistant phenotypes, this approach illustrates how microfluidic technologies can evolve into data-driven diagnostic systems capable of informing personalized antimicrobial therapy.

## Methods

The droplet microfluidic device fabrication with the ASIGA 3D printer and overall design, which consists of droplet generation and storage, is similar to that previously described^46^. The continuous phase consisted of Novec HFE-7500 engineering fluid (3 M, USA) supplemented with 2% (w/w) FluoSurf-C surfactant (Emulseo, France). The dispersed phase was prepared using bacterial culture medium containing the antibiotic of interest and diluted overnight bacterial cultures adjusted to the required concentrations. Fluids were infused into the microfluidic chip using syringe pumps (Nemesys, Cetoni, Germany). The continuous phase and the dispersed bacterial suspension were loaded in separate BD syringes (1 ml volume) and connected to the two separate inlets. Typical flow rates maintained were 4 μL/min for the continuous phase and 1 μL/min for the dispersed phase, which resulted in monodispersed droplet generation. The microfluidic device was mounted on a stage-top incubator (OKOlab) and maintained at 37 °C and 80% relative humidity to ensure steady conditions for bacterial growth within droplets. The microfluidic chip with inlets and outlets was placed inside a stage-top incubator and mounted on an inverted microscope (IX73, Olympus, Japan). Imaging was performed using a 10× objective, a motorized x–y stage, and a camera (C11440, Hamamatsu, Japan). Experiments with *K. pneumoniae*, *P. aeruginosa*, and *A. baumannii* were conducted for 24 h with image acquisition every 2 h. For *S. aureus*, experiments were conducted for 48 h with image acquisition every 3 h. Imaging positions were pre-marked using the motorized stage, and images were acquired at the defined intervals.

Bacterial strains frozen in 10% DMSO at −80 °C were picked up on Mueller-Hinton (MH) agar plates and incubated overnight at 37 °C. Single colonies were added to 1 ml MH broth and incubated overnight at 37 °C, shaking, before each experiment. For *S. aureus*, brain-heart infusion (BHI) was used for liquid cultures and microfluidic experiments, and MH broth was used for other species. Amikacin, meropenem, and vancomycin were purchased from Sigma-Aldrich and dissolved in either sterile water or DMSO before use. Meropenem was prepared fresh before each experiment, and amikacin and vancomycin were frozen in single-use aliquots at −20 °C. More information about the strains, resistance mechanisms, and MICs is provided in Table 7. Additional information on strain origin is provided in the Supplementary Table 2.

**Table 7:** Strain information

Strain	Species	Comment	The resistance mechanism involved in HR	Antibiotic & MIC (mg/L)
DA69806	*P. aeruginosa*	HR MER		MER, 2
DA69786	*P. aeruginosa*	Non-HR MER	N/A	MER, 0.25
DA33098	*A. baumannii*	HR AMK	*mdh* ∆9 nt^14^	AMK, 12
DA33414	*A. baumannii*	Non-HR AMK	N/A	AMK, 2
DA33140	*K. pneumoniae*	HR AMK	Aac(6´)-lb-cr5^15^	AMK, 3
DA33145	*K. pneumoniae*	Non-HR AMK	N/A	AMK, 1
DA75526	*S. aureus*	Mu3, hVISA (HR VAN)		VAN, 2
DA70300	*S. aureus*	Non-HR VAN	N/A	VAN, 1

In PAP-based definitions of HR, resistant subpopulations are identified by their ability to survive and grow at antibiotic concentrations several-fold higher than the minimum inhibitory concentration (MIC) of the dominant population, typically at least four-fold above the clinical breakpoint or modal MIC. The antibiotic concentrations used in our droplet-based assays were selected to be consistent with this conceptual framework rather than to replicate breakpoint testing per se. Specifically, for each species–antibiotic pair, concentrations were chosen that exceeded the MIC of the dominant susceptible population and that, based on prior PAP characterization of the same strains, selectively permitted growth of known HR subpopulations while fully inhibiting the bulk population. This mirrors the PAP principle of applying supra-inhibitory antibiotic pressure to reveal rare resistant cells, but implements it in a confined, planktonic microenvironment rather than on solid agar.

We note that the use of a single supra-inhibitory concentration in the droplet assay is intentional. Unlike PAP, which relies on a full concentration gradient to reconstruct population survival curves, the objective of the present platform is to detect and estimate the frequencies of HR subpopulations under selective pressure. Using a concentration well above the MIC of the susceptible population maximizes contrast between growth-positive and growth-negative droplets and enables robust frequency estimation without requiring multiple parallel concentrations.

Images were processed as previously described^46^. Briefly, images were smoothened, morphologically opened, min-max-normalized, and thresholded using Otsu’s method to identify droplet boundaries (parameters as originally published). Droplets were then segmented using a circle Hough transform and a centroid-seeded Watershed algorithm as previously described, discarding droplets touching the image border. Extracted droplet sizes were normalized by the average diameter at 0 h for downstream analysis. Additionally, images were further processed to quantify droplet texture. For each segmented droplet, a centered, square-shaped patch fitting the droplet’s rectangular bounding box was extracted. Gray-Level Co-occurrence Matrices (GLCMs) were computed for all patches, quantifying how often different combinations of pixel intensity values occur within the respective patch. To calculate the GLCM, pixel pairs were defined as being in the same row and 4 pixels apart. The GLCMs were then used to calculate contrast, dissimilarity, homogeneity, and correlation values for each droplet using scikit-image v0.23.2^55^. Of these properties, texture homogeneity and correlation were chosen to distinguish between empty and filled droplets as they provided highly consistent and significant results of the different features evaluated. Examples of the texture feature involving heatmaps of homogeneity and correlation are shown in Supplementary Figs. 3 and 4.

Specifically, for each experiment, we extracted the texture features (homogeneity and correlation) from droplets at 0 h (6 h for *P. aeruginosa*), when bacterial growth had not yet occurred. We then defined the cutoff criterion using the lowest (most conservative) baseline value of these metrics observed at 0 h in that experiment. This baseline-derived cutoff was subsequently applied to classify droplets at the 24- or 48-h time point to estimate the frequency of the resistant subpopulation. The rationale for using this criterion is that the 0-hour droplet population represents the true no-growth reference state under the same imaging conditions as the subsequent timepoints. Therefore, using an experiment-specific baseline cutoff provides a normalization strategy and minimizes false-positive growth classification from imaging artifacts. This is particularly important in our application because any false-positive droplets would directly inflate the estimated resistant subpopulation frequency. This image process could be applied easily for a new antibiotic-bacteria combination by following these steps: (1) Run a baseline calibration experiment for a specific strain. (2) Extract texture features at 0 h to define the no-growth condition. (3) Define the cutoff threshold. (4) Validate. This workflow requires only a short baseline acquisition at 0 h and does not require training a classifier or building a large dataset, making it practical for broad adoption and rapid extension to new bacteria–antibiotic pairs.

## Supplementary information

Supplementary Information

## Data Availability

All the raw images used for analysis are available on Figshare. S.N.A., N.F.K., E.V., J.W., A.A.C., D.I.A., and M.T. Droplet microfluidics with image texture quantification for the detection of rare antibiotic-resistant subpopulations from the bloodstream infections dataset. Figshare (10.17044/scilifelab.31452181), and the code used for the analysis is on Zenodo (10.5281/zenodo.18717330).
