# Supplementary material for: Droplet microfluidics with image texture quantification for detection of rare antibiotic-resistant subpopulations from bloodstream infections
Source: NPJ Digit Med. 2026 May 30;9:410. doi: 10.1038/s41746-026-02808-x (PMC13222359; doi:10.1038/s41746-026-02808-x)
Supplement: Supplementary file 1 — Supplementary Information [file 41746_2026_2808_MOESM1_ESM.pdf]

Supplementary Information

of

Droplet microfluidics with image texture quantification for detection of rare antibiotic-resistant subpopulations from bloodstream infections

Sagar N. Agnihotri [a,b,c], Nikos Fatsis-Kavalopoulos [c], Emma Vikdahl [c], Jonas Windhager [d], Agustin A. Corbat [e], Dan I. Andersson [c*], and Maria Tenje [a,b*]

[a].Department of Materials Science and Engineering, Uppsala University, Uppsala, Sweden

[b] Science for Life Laboratory, Uppsala University, Uppsala, Sweden

[c] Department of Medical Biochemistry and Microbiology, Uppsala University, Uppsala, Sweden

[d] Department of Information Technology and SciLifeLab BioImage Informatics Unit, Uppsala University, Uppsala, Sweden

[e] Instituto de Física de Buenos Aires, Departamento de Física, Facultad de Ciencias Exactas y Naturales, Universidad de Buenos Aires, Argentina.

[*]Corresponding author: maria.tenje@angstrom.uu.se and dan.andersson@imbim.uu.se

Supplementary Table 1: The data showing details about each experiment conducted, which includes droplet content, average droplet diameter and volume and bacterial encapsulation per droplet.

Droplet content	Experiment number	Average diameter (µm)	Average volume (nL)	Bacteria encapsulation per droplet
Acinetobacter baumannii				
Non-HR strain DA33414 (5×10^8 CFU/mL) in MH broth + 24 mg/L Amikacin	N_1	218	5.42	2710
	N_2	214	5.13	2565
	N_3	211	4.92	2460
HR strain DA33098 (5×10^8 CFU/mL) in MH broth + 24 mg/L Amikacin	N_1	214	5.13	2565
	N_2	212	4.99	2495
	N_3	209	4.78	2390
Klebsiella Pneumoniae				

Non-HR strain DA33145 (5×10[8] CFU/mL) in MH broth + 12 mg/L Amikacin	N_1	218	5.42	2710
	N_2	216	5.28	2640
	N_3	215	5.20	2600
HR strain DA33140 (5×10[8] CFU/mL) in MH broth + 12 mg/L Amikacin	N_1	210	4.85	2425
	N_2	191	3.65	1825
	N_3	191	3.65	1825
Pseudomonas Aeruginosa				
Non-HR strain DA69786 (10[8] CFU/mL) in MH broth + 8 mg/L Meropenem	N_1	211	4.92	492
	N_2	207	4.64	464
	N_3	212	4.99	499
HR strain DA69806 (10[8] CFU/mL) in MH broth + 8 mg/L Meropenem	N_1	217	5.35	435
	N_2	216	5.28	528
	N_3	216	5.28	528
Staphylococcus Aureus				
Non-HR strain DA70300 (5×10[8] CFU/mL) in BHI media + 8 mg/L Vancomycin	N_1	193	3.76	1880
	N_2	196	3.94	1970
	N_3	199	4.13	2065
HR strain DA75526 (5×10[8] CFU/mL) in BHI media + 8 mg/L Vancomycin	N_1	204	4.45	2225
	N_2	202	4.32	2160
	N_3	202	4.32	2160

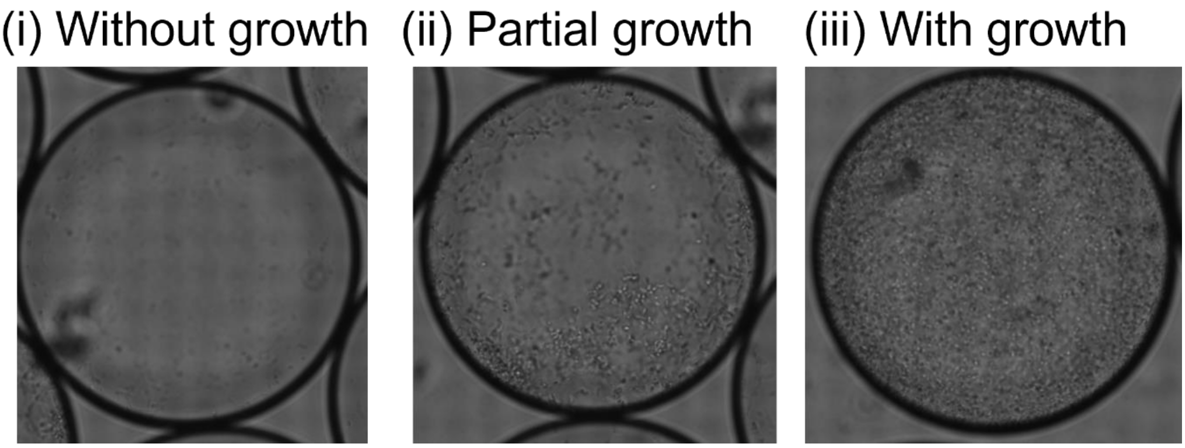

Supplementary Figure 1: At 24 hours, zoomed in view of (i) droplet showing no growth of bacteria. (ii) Droplet showing partial growth of bacteria. (iii) Droplet showing full growth of bacteria.

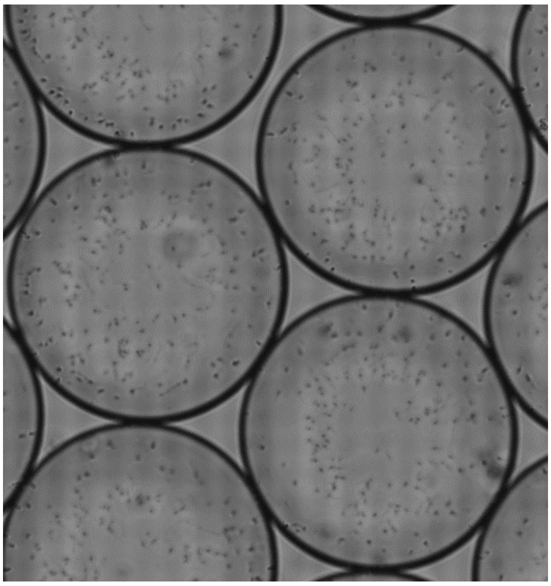

Supplementary Figure 2: Zoomed-in view showing droplets without *P. aeruginosa* without growth in the presence of Meropenem. After the antibiotic action, small dot-like residual structures appeared, likely corresponding to bacterial debris or lysed cells, which in turn reduced the correlation feature. The image shown is at 6 hours after the start of imaging.

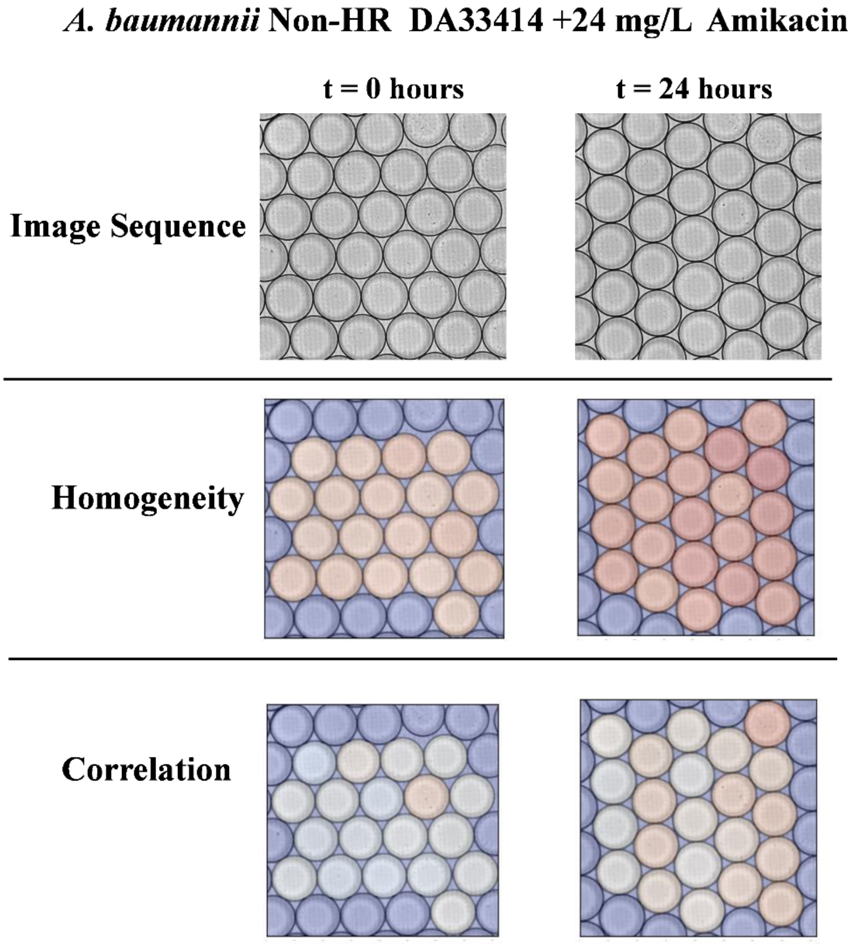

Supplementary Figure 3: Representative examples of all non-growing droplets with corresponding texture analysis features for homogeneity and correlation. Top row: Optical microscopy images of non-HR *A. baumannii* droplet arrays at 0 hours (left) and 24 hours (right) following exposure to 24 mg/L

amikacin. Middle and bottom rows: heatmaps showing texture homogeneity and correlation at 0 and 24 hours.

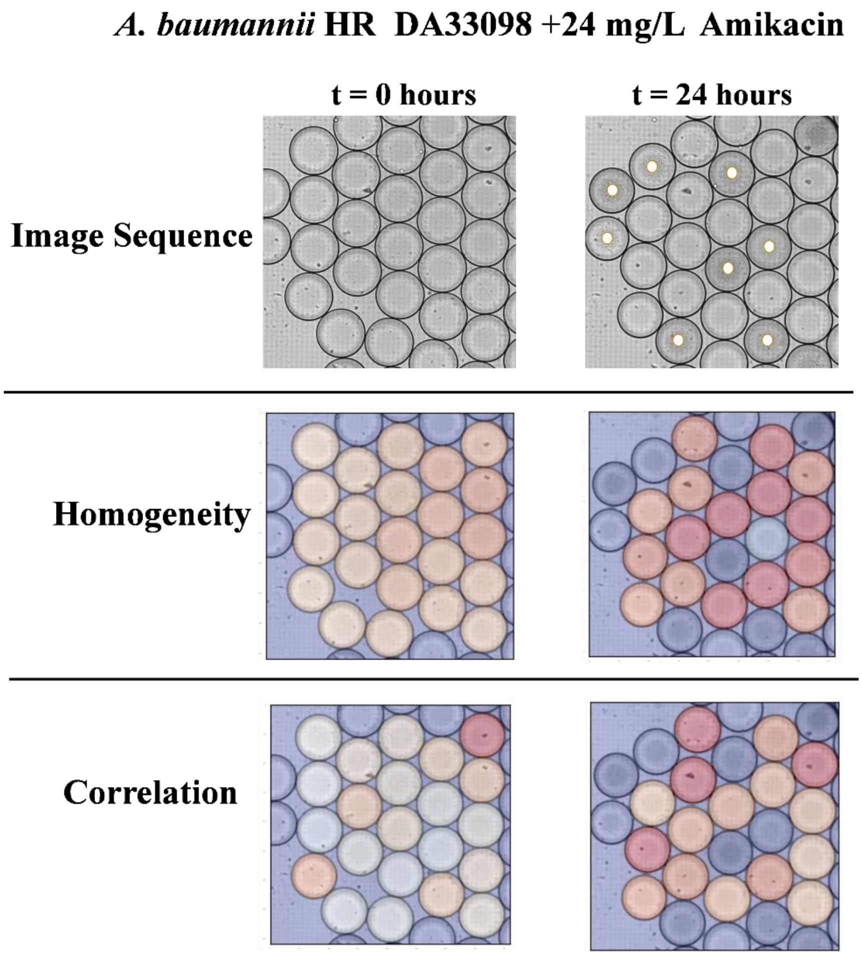

Supplementary Figure 4: Representative examples of growing and non-growing droplets with corresponding texture analysis features for homogeneity and correlation. Top row: Optical microscopy images of HR *A. baumannii* droplets at 0 hours (left) and 24 hours (right) following exposure to 24 mg/L amikacin, with white dots indicating droplets exhibiting growth. The middle and bottom rows show heatmaps showing texture homogeneity and correlation at 0 and 24 hours, respectively.

Supplementary Note 1

Calculation for experimentally calculated subpopulation frequencies using texture quantification:

Encapsulation of both Gram-negative and Gram-positive bacteria in droplets is governed by the Poisson distribution and depends on the initial concentration of bacteria (CFU/mL) and the volume of the droplet. The detailed calculations are as follows: When a 5×10^8 CFU/mL of a particular bacterial species in MH broth or BHI media is compartmentalized in a droplet of 5.42 nL volume, the average number of CFU per droplet (λ) can be calculated as:

$$\lambda = \frac{5 \times 10^8\, CFU}{mL} \times \frac{5.42\, nL}{droplet} = 2710\, CFU/droplet$$

The CFU/droplet obtained is then used to calculate the experimental HR frequency as follows:

$$Experimental\ HR\ frequency = \frac{N_G}{N_d \times \lambda}$$

N_G is the number of droplets showing bacterial growth according to the cutoff criteria, while N_d is the number of droplets analyzed.

Supplementary Table 2: Additional strain information table.

Strain	Species	Strain origin
DA69806	*P. aeruginosa*	Clinical strain, provided by Soren Molin and Helle Krogh Johansen from DTU.
DA69786	*P. aeruginosa*	Clinical strain, provided by Soren Molin and Helle Krogh Johansen from DTU.
DA33098	*A. baumannii*	Clinical strain provided by Felipe Cuenca[1]
DA33414	*A. baumannii*	Clinical strain provided by Cheng-Hsun Chiu[2]
DA33140	*K. pneumoniae*	Clinical isolate from the Public Health Agency, Sweden[3]
DA33145	*K. pneumoniae*	ATCC700603, ESBL reference strain
DA75526	*S. aureus*	Mu3 (hVISA), received from Sarah Satola, Emory University School of Medicine, Atlanta
DA70300	*S. aureus*	Clinical isolate from Rafael Cantón, Hospital Universitario Ramón y Cajal, Madrid, Spain

Supplementary References:

1. Fernández Cuenca, F. *et al.* Prevalence and analysis of microbiological factors associated with phenotypic heterogeneous resistance to carbapenems in Acinetobacter baumannii. *Int. J. Antimicrob. Agents* **39**, 472–477 (2012).

2. Lee, H.-Y. *et al.* Imipenem heteroresistance induced by imipenem in multidrug-resistant Acinetobacter baumannii: mechanism and clinical implications. *Int. J. Antimicrob. Agents* **37**, 302–308 (2011).

3. Nicoloff, H., Hjort, K., Andersson, D. I. & Wang, H. Three concurrent mechanisms generate gene copy number variation and transient antibiotic heteroresistance. *Nat. Commun.* **15**, 3981 (2024).
